# Malignancies diagnosed before and after anal squamous cell carcinomas: A SEER registry analysis

**DOI:** 10.1002/cam4.3909

**Published:** 2021-05-07

**Authors:** Krupa S. Jani, Shou‐En Lu, James D. Murphy, Paul B. Romesser, Krishan R. Jethwa, Diana Li, Anupama Chundury, Abraham J. Wu, Lara Hathout, Christopher L. Hallemeier, Salma K. Jabbour

**Affiliations:** ^1^ Department of Radiation Oncology Rutgers Cancer Institute of New Jersey Rutgers Robert Wood Johnson Medical School Rutgers University New Brunswick NJ USA; ^2^ Department of Biostatistics School of Public Health Rutgers University Piscataway NJ USA; ^3^ Department of Radiation Medicine and Applied Sciences Altman Clinical Translational Research Institute University of California San Diego San Diego CA USA; ^4^ Department of Radiation Oncology Memorial Sloan Kettering Cancer Center New York NY USA; ^5^ Department of Therapeutic Radiology Yale University New Haven CT USA; ^6^ Department of Radiation Oncology Mayo Clinic Rochester MN USA

**Keywords:** anus neoplasms; neoplasms, second primary, SEER program

## Abstract

**Background:**

Increased risk of a second primary malignancy (SPM) before or after diagnosis of anal squamous cell carcinoma (ASCC) has been reported in a previous single‐institution study. We hypothesize that patients diagnosed with ASCC are at increased risk for developing SPMs before or after the diagnosis of ASCC. The primary objective of this study was to identify the diagnoses of cancer most likely to occur as SPMs before or after ASCC.

**Methods:**

This work employs the Surveillance, Epidemiology, and End Results (SEER) Program registry data to conduct a US‐population‐based study of patients diagnosed with ASCC between 1975 and 2016. In patients diagnosed with ASCC, we evaluated the risk of SPMs and the risk of developing ASCC as an SPM after another cancer using standardized incidence ratios (SIR) for all SPMs by calculating the ratio of observed events in the ASCC cohort compared to expected (O/E) events in a matched reference cohort of the general population.

**Results:**

A total of 7,594 patients with primary ASCC were included. Patients with ASCC were at increased risk of the diagnosis of an SPM (SIR = 1.45), particularly cancers of the lung, vulva, oropharynx, or colon. Patients with ASCC had an increased rate of previous malignancy (SIR = 1.23), especially Kaposi sarcoma or vulvar cancer. Overall elevated incidence of SPMs was unrelated to prior radiation treatment. Radiation treatment was associated with increased risk for SPMs in the female genital system but appeared protective against prostate cancer as SPMs.

**Conclusions:**

Our findings support increased surveillance and screening for second malignancies in patients with these diagnoses, as patients with ASCC are often either survivors of a prior cancer diagnosis or are at increased risk of developing later malignancies.

## Graphical abstract

Survivors of anal squamous cell carcinoma are at increased risk for particular second malignancies and should be screened for these. Risk of second malignancies is sometimes related to radiation therapy, but radiation can also be protective against second cancers in some cases.

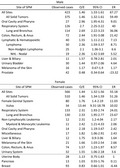

## 1 INTRODUCTION

Improvements in cancer screening, as well as early intervention, have led to falling rates of cancer deaths in the United States over the past two decades.^1^ Owing to these successes, survivorship in cancer patients increased and resulted in a significant population of cancer survivors—approximately 16.9 million at the beginning of 2019 in the United States; due to the growth and aging of the population, it is estimated that this number will rise to 22.1 million by 2030.^2^ Cancer survivors often undergo lifelong surveillance and increased screening for further malignancies since they are predisposed to developing new cancers due to a variety of risk factors such as genetic disorders, somatic mutations, DNA‐damaging treatment‐induced mutations, environmental exposures, or other treatment‐related changes.^3^, ^4^ Indeed, 17–19% of patients who present with new cancers are survivors of previous malignancies.^5^

Anal cancer is a rare malignancy, with 8,590 new cases estimated in 2020 and 1,350 estimated deaths.^1^ The most common histologic type of anal cancer is anal squamous cell carcinoma (ASCC). Infection with human papillomavirus (HPV) is associated with over 90% of cases.^6^ A recently published U.S. population‐based study concluded that incidence and mortality rates of ASCC continue to rise, with incidence increasing at a rate of 2.7% per year.^7^ The mainstay of treatment for patients with localized, non‐metastatic anal cancer consists of definitive concurrent radiotherapy with mitomycin‐C and 5‐fluorouracil as originally established by Nigro et al.^8^ A single‐institution study of 46 patients suggested that individuals diagnosed with ASCC who were treated with chemoradiation are at increased risk for diagnosis with an SPM either before or after their anal cancer diagnosis. The incidences of these second malignancies appear unrelated to the risk of secondary malignancy associated with radiation therapy delivery.^9^

Patients diagnosed with ASCC are at particularly high risk of developing second cancers. About 50% of patients with ASCC present with localized disease, for which the 5‐year relative survival rate is approximately 80% for early stage disease.^10^ The risk for developing second cancer may be related to the association of ASCC with HPV infection^6^, ^10^ and a previous study reported an increased rate of developing an HPV‐related second primary malignancy (SPM) in patients diagnosed with ASCC.^11^

Using the U.S. Surveillance, Epidemiology, and End Results (SEER) Program registry, we conducted a U.S. population‐based study that included patients diagnosed with ASCC to evaluate the incidence of SPM either before or after the diagnosis of ASCC. We hypothesized that patients may experience an intrinsic increased risk of cancer associated with ASCC, unrelated to treatment, and aimed to determine the specific incidence of second cancers in this patient population. The primary objective of this study was to identify the diagnoses of cancer most likely to occur as SPMs before or after the diagnosis of ASCC.

## 2 METHODS

### 2.1 Source dataset

The study population was identified using recorded data in the U.S. SEER Program of the National Cancer Institute.^12^ Data on patient demographics, primary tumor site, tumor morphology, staging, first treatment course, and survival follow‐up are collected routinely as part of the SEER registries. Our cohorts were generated using data from the SEER 9 Registries November 2018 submission using cancers diagnosed between 1975 and 2016 (n = 3,600,102 for the entire registry) which covers approximately 9.4% of the U.S. population.

In general, multiple primary malignancies are reported in the SEER dataset and are defined according to coding rules which include multiple sites as well as different histology or morphology groupings to avoid the misclassification of metastases or multifocal tumors as multiple primaries. This grouping is also done to prevent the misclassification of recurrences as SPMs, for example, in patients with a first diagnosis of ASCC who then had a second diagnosis of ASCC. SPMs are grouped within organ systems and reported by anatomic location in the SEER registry. For example, SPMs may be reported in the “Female Genital System” through SEER, a classification that includes the uterine cervix, uterine body, ovary, vagina, and vulva.

### 2.2 Patient cohorts

Using the SEER Registries dataset, we generated two cohorts of patients: 1) in patients diagnosed with ASCC who survived for a minimum of 2 months, we evaluated the risk of subsequent cancers and 2) we evaluated the risk of developing ASCC as a second primary after any other cancer. In both cohorts 1 and 2, we only considered malignancies outside a 2‐month window following the initial cancer diagnosis, in accordance with recommendations by the SEER database to distinguish between synchronous and metachronous multiple primaries as this study focuses on metachronous primary cancers.

Cohort 1 for this analysis consists of individuals diagnosed first with primary ASCC and who developed an SPM after treatment of ASCC. This cohort was generated by including patients using squamous cell carcinoma histology codes 8050–8089 together with site codes (ICD‐O‐3/WHO 2008) for “Anus, Anal Canal and Anorectum.” Cases of SPMs that were diagnosed at autopsy or lost to follow‐up were excluded from the analysis. Data on whether these patients received external beam radiation therapy for their index cancer of ASCC were also collected.

In cohort 2, data for patients with ASCC diagnosed as an SPM in patients diagnosed previously with any other cancer were collected by the above histology codes to filter results for second malignancies within each cancer histologic subtype provided in the registry.

### 2.3 Statistical analysis

The increased risk of a secondary cancer was quantified with standardized incidence ratios (SIR), which were defined as the ratio of observed to expected (O/E) second cancers. The referent rate for the expected number of cancers was calculated for a reference cohort among the general population matched for age, gender, race, and year of diagnosis. Excess risk (ER) values representing excess cancers beyond the expected amount were reported per 10,000 persons per year. We reported confidence intervals (CI’s) as 95% confidence intervals. All analyses were conducted with statistical program SEER∗Stat version 8.3.5 provided by the National Cancer Institute utilizing the multiple primary standardized incidence ratio (MP‐SIR) tool.^13^, ^14^ SIR values are not presented when the number of observed cancers was less than 11 cases. SIR values are presented for cancers with observed values larger than 11 by grouping within the organ system when appropriate. Results reported within each table include all significant SIRs from highest to lowest within each subtype. Selected non‐significant results are included in tables (in the Results section) separated by a dashed line as a basis for comparison.

## 3 RESULTS

### 3.1 Cohort 1 (patients with primary ASCC followed by SPM)

Cohort 1 consisted of all men and women diagnosed with primary ASCC included in the SEER database between 1975 and 2016 (n = 7,594). Of these, 989 (13%) subsequently developed SPMs, in comparison to an expected number in the reference population of 68, for a SIR of 1.45 (95% CI: 1.36–1.54).

In Cohort 1, SIR values for SPMs were elevated overall, in all solid tumors, and in oral cavity and pharynx, digestive system, colon/rectum/anus, and the respiratory system in both men and women. Incidence for SPMs demonstrated an elevated number of cases in men for lymphatic and hematopoietic diseases (SIR = 1.55; ER = 7.13), including lymphoma and non‐Hodgkin lymphoma. In women, an increased incidence of SPMs including skin melanomas (SIR = 1.66; ER = 2.66), female genital system (SIR = 1.76, ER = 11.03), urinary system (SIR = 1.55; ER = 3.6), and leukemias (SIRs ranged from 1.82 to 2.42; ERs between 2.06 and 2.17) were observed (Table 1).

**TABLE 1:**
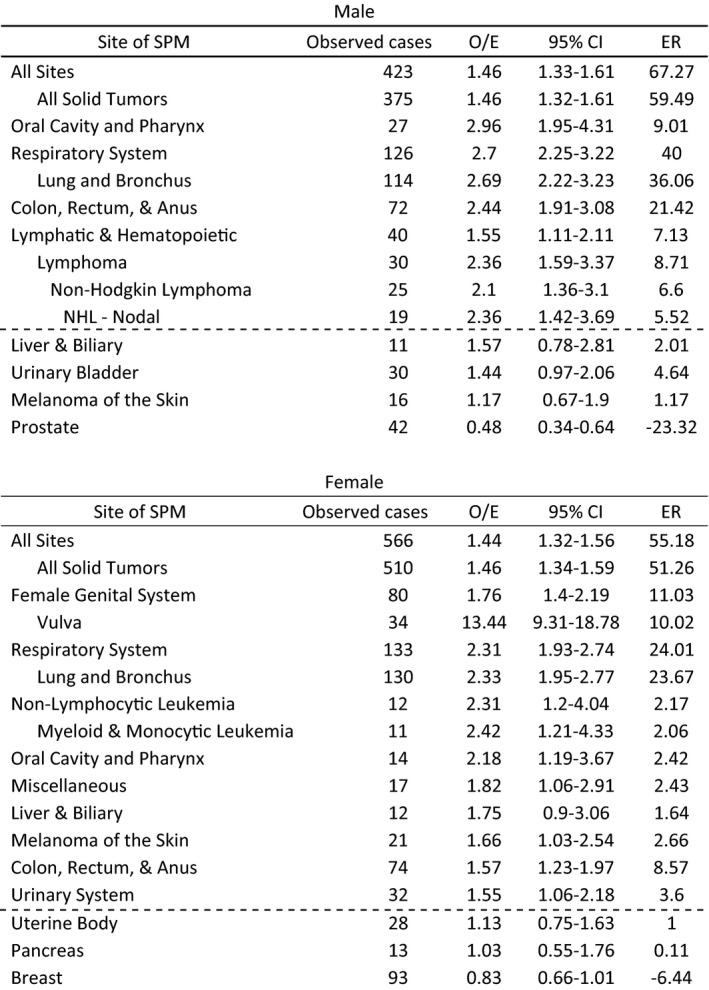
Incidence of selected second primary malignancies in men and women diagnosed with index primary anal squamous cell carcinomas, arranged by standardized incidence ratios (SIR) from highest to lowest within each subtype. Selected non‐significant results are reported below, separated by a dashed line. Observed number of cases, observed‐to‐expected ratio (O/E), 95% confidence interval (CI), and excess risk (ER) are reported

In Cohort 1, a high excess risk of second primary diagnosis of lung cancer after ASCC was observed in both male (SIR = 2.69; ER = 36.506 and female (SIR = 2.33; ER = 23.67) patients. For males, the increased excess risk for SPM was associated with a second cancer of the colon/rectum/anus (SIR = 2.44; ER = 21.42) and with oral cavity or pharyngeal cancer (SIR = 2.96; ER = 9.01). In females, vulvar cancer carried strongly increased risk (SIR = 13.44; ER = 10.02) as well as malignancies in the rectum and anus (SIR = 3.56; ER = 9.85) as per SEER coding rules for SPMs.

### 3.2 Cohort 2 (another primary malignancy followed by ASCC as an SPM)

Cohort 2 consisted of 1,297 patients (599 men and 698 women) with a diagnosis of ASCC as an SPM after a different first primary malignancy diagnosis, representing 17% of patients ever diagnosed with ASCC (Table 2). This frequency also exceeds the expected number based on the reference population (expected = 1,054) with SIR = 1.23 (95% CI: 1.16–1.3).

**TABLE 2:**
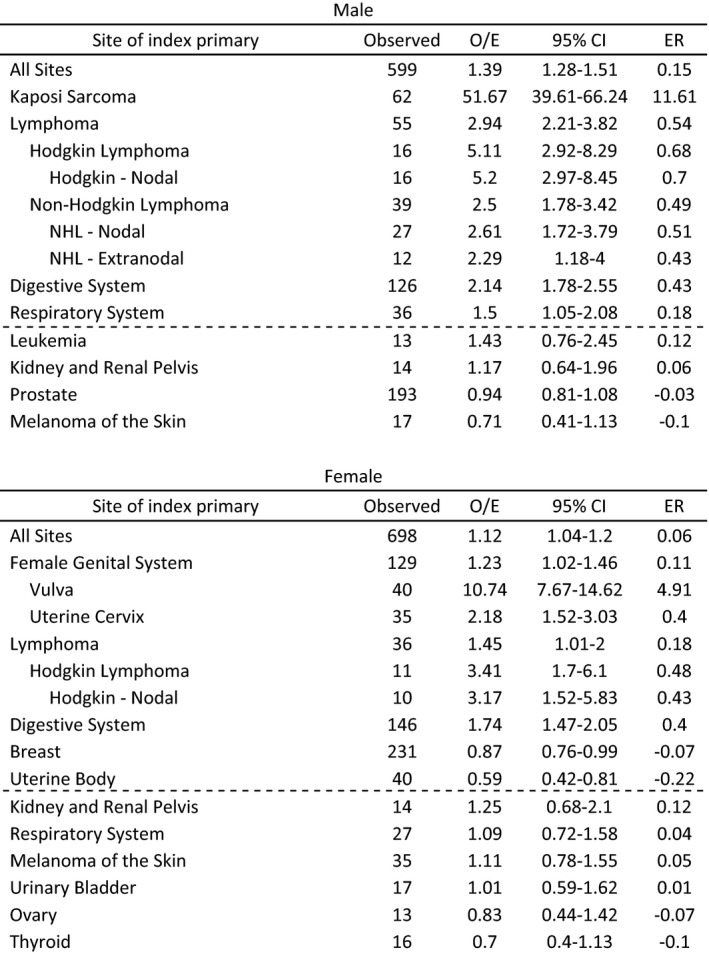
Incidence of anal squamous cell carcinoma as a second primary malignancy in patients with another index primary cancer by the site of first primary malignancy, organized from highest to lowest SIR within each subtype. Selected non‐significant results are reported below, separated by a dashed line. Observed number of cases, observed‐to‐expected ratio (O/E), 95% confidence interval (CI), and excess risk (ER) are reported

Cancers associated with an increased risk of subsequent ASCC diagnosis included all sites, digestive system, and lymphoma in males and females. In males, an additional increased risk of a later ASCC diagnosis was associated with an index diagnosis of Kaposi sarcoma (SIR = 51.67; ER = 11.61). In females, increased risk for ASCC as an SPM was associated with the first primary diagnosis of vulvar cancer (SIR = 10.74; ER = 4.91). Women with a first primary diagnosis of breast or uterine body cancers were at a slightly decreased risk for ASCC as an SPM (SIR = 0.87 and 0.59; ER = −0.07 and −0.22, respectively).

### 3.3 Prior therapies

In Cohort 1, most patients received definitive radiation therapy in the treatment of their ASCC: in total, 718 (75%) patients from Cohort 1 (with SPMs after ASCC) underwent treatment with external beam radiation therapy (EBRT), while 238 patients with SPMs did not receive radiation therapy. To evaluate the relationship between radiation therapy for index primary ASCC and later SPMs, we compared the SIR values for all SPMs in patients who were treated with radiation therapy to SIR values for those not treated with radiation. In this cohort, 273 male and 445 female patients with SPMs had been treated with radiation, while 140 male and 98 female patients did not receive radiation but developed SPMs.

For most sites of SPMs in all patients in this cohort, we found that the overall elevated incidence of SPM was unrelated to whether EBRT had been delivered previously for ASCC (Table 3, All Patients). EBRT was associated with increased risk for certain SPMs in female patients treated for a first primary ASCC (Table 3), specifically in cancers of the female genital system (SIR = 1.84; ER = 12.11). In men, the treatment of ASCC with EBRT appeared to be protective against SPMs (Table 3), specifically with prostate cancer as an SPM in ASCC patients treated with EBRT (SIR = 0.37; ER = −28.31). No significant difference in SIR for an SPM of prostate cancer was observed in patients with ASCC who did not receive EBRT. In patients with ASCC who did not receive EBRT, we observed increased incidences of urinary bladder cancer (SIR = 2.13; ER = 11.93) as well as lymphatic and hematopoietic diseases (SIR = 1.9; ER = 11.46); however, we did not observe similar increases in patients who did receive EBRT for ASCC.

**TABLE 3:** Incidence of second primary malignancies in patients treated with or without external beam radiation therapy (EBRT) for first primary ASCC. Observed number of cases, observed‐to‐expected ratio (O/E), 95% confidence interval (CI), and excess risk (ER) are reported

	All Patients							
	No radiation or unknown				EBRT			
	Observed	O/E	95% CI	ER	Observed	O/E	95% CI	ER
All sites	238	1.49	1.3–1.69	66.24	718	1.44	1.33–1.54	57.76
*Male*								
Prostate	18	0.72	0.43–1.14	−12.13	23	0.37	0.24–0.56	−28.31
Urinary bladder	13	2.13	1.13–3.64	11.93	17	1.19	0.7–1.91	2.04
Lymphatic & hematopoietic	14	1.9	1.04–3.18	11.46	25	1.4	0.9–2.06	5.23
*Female*								
Female genital system	12	1.36	0.7–2.37	5.28	64	1.84	1.42–2.35	12.11

### 3.4 Timing of second primary malignancy

**FIGURE 1:**
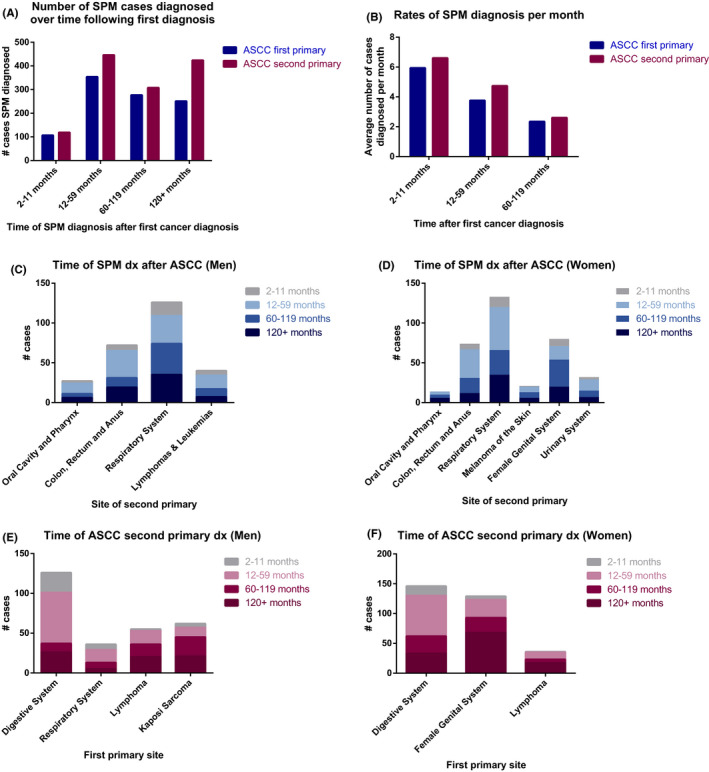
Temporal distribution of secondary cancer diagnoses relative to anal squamous cell carcinoma diagnosis. (A) Total number of all observed second primary malignancy cases within 1 year, 1–5 years, 5–10 years, or more than 10 years after first cancer. Blue indicates cases in Cohort 1, diagnosed after an index diagnosis of ASCC, while red indicates cases from Cohort 2 in which ASCC was the second primary malignancy diagnosed after another previous cancer. (B) Rates of SPM diagnoses showing the average number of diagnosed SPM cases per month in ASCC patients with second cancer diagnoses. (C‐D) Number of second primary cases diagnosed following the first diagnosis of ASCC in men and women organized by selected sites of the second primary cancer and timing of diagnosis following ASCC diagnosis. (E‐F) Number of ASCC cases diagnosed as a second primary malignancy in patients with another prior cancer organized by selected sites of first primary and timing of ASCC diagnosis

We examined the temporal distribution of when SPMs were diagnosed by organizing the timing of SPM diagnosis into the following categories from time of first cancer diagnosis: 2–11 months, 12–59 months, 60–119 months, and 120+ months. The observed number of SPM cases was greatest in the period between 12 and 59 months after diagnosis of the first primary in all patients (Figure 1A, Table 4). Furthermore, we also noted a bimodal distribution of total observed ASCC cases diagnosed between 1–5 years and >10 years following a previous cancer diagnosis in Cohort 2 (Figure 1A). However, data analyzing the O/E values over time suggest that patients are at risk for different SPM cancer types at different time periods following their initial cancer diagnosis (Table 4). While the overall rate of new cases diagnosed per month was greatest in the first year (Figure 1B), the O/E value was significantly elevated at time periods beyond one year after initial diagnosis.

**TABLE 4:** SIR (O/E) values of second primary malignancies in patients according to time in months since diagnosis of index malignancy. Significant O/E values are marked with *

Cohort 1: ASCC index malignancy					
Site of second malignancy	2–11 months	11–59 months	60–119 months	120+ months	Overall
*Male*					
All sites	1.94*	1.62*	1.47*	1.18	1.46*
Oral cavity & pharynx	3.3	4.26*	2.03	2.23	2.96*
Colon, rectum, and anus	2.55*	3.63*	1.51	2.01*	2.44*
Respiratory system	3.98*	2.39*	3.13*	2.29*	2.70*
Lymphatic & hematopoietic	2.55	2.13*	1.46*	0.81	1.55*
*Female*					
All sites	1.46*	1.56*	1.56*	1.20*	1.44*
Oral cavity and pharynx	0	1.82	2.35	3.21*	2.18*
Colon, rectum and anus	1.54	2.30*	1.53	0.83	1.57*
Respiratory system	2.45*	2.86*	2.02*	1.93*	2.31*
Melanoma of the skin	0.79	1.59	2.08	1.66	1.66*
female genital system	1.92	1.06	2.80*	1.6	1.76*
Urinary system	1.56	2.06*	1.46	1.07	1.55*

Cohort 2: ASCC SPM					
Site of first malignancy	2–11 months	11–59 months	60–119 months	120+ months	Overall
*Male*					
All sites	1.63*	1.43*	1.29*	1.37*	1.39*
Digestive system	3.44*	3.16*	0.76	1.53	2.14*
Respiratory system	1.44	1.81*	1.6	0.96	1.50*
Lymphoma	1.13	2.84*	3.44*	3.16*	2.94*
Kaposi sarcoma	46.10*	35.56*	81.43*	45.73*	51.67*
*Female*					
All sites	0.96	1.35*	1.04	1.03	1.12*
Digestive system	1.74	2.50*	1.39	1.25	1.74*
Breast	0.78	0.96	0.93	0.78*	0.87*
Female genital system	0.85	1.17	1.06	1.39*	1.23*
Lymphoma	0.45	1.54	0.95	1.99*	1.45*

Of the SPMs diagnosed in patients from Cohort 1 over the first year, lung and GI cancers predominated. However, we observed that of all the lung and GI cancers diagnosed as SPMs, most cases of second primary lung cancer in both men and women were diagnosed after the first year, with a slightly higher proportion of cases in the period 1–5 years after ASCC diagnosis in women compared to men (Figure 1C‐D). Although the absolute number of cases drops after 5 years, the SIR remains significantly elevated for lung cancer in both men and women long‐term, even after ≥10 years following the initial cancer diagnosis (Table 4). Most SPM cases of oropharyngeal cancer or female genital cancers were not diagnosed in the first year, and the risk for SPMs at these sites increased only at later times.

In Cohort 2 (patients with ASCC as the SPM), we found that this second diagnosis sometimes occurred notably later than the first cancer diagnosis, and was often made 10+ years after the initial primary cancer (Figure 1A,E‐F; Table 4). In men with a first primary cancer diagnosis of lung cancer, for example, most ASCC cases were diagnosed between 1 and 5 years later. In men diagnosed with lymphoma, almost all subsequent ASCC diagnoses took place after the first year. Men who were first diagnosed with Kaposi sarcoma had more cases of ASCC diagnosed 5 or more years later rather than sooner, although a significant incidence ratio was found in the first year as well (Table 4). Women with female genital system cancers had ASCC diagnosed much later, with the majority of ASCC cases diagnosed 10+ years after (Table 4).

## 4 DISCUSSION

Patients diagnosed with ASCC experienced multiple primary malignancies at an increased rate. In this large‐scale SEER population study, we identified two cohorts of patients who had been diagnosed with ASCC and who appeared to be at increased risk for SPMs either before or after the diagnosis of ASCC. In Cohort 1 (patients first diagnosed with ASCC), the overall SIR for both men and women to develop an SPM at any site was 1.45, suggesting a 45% increased relative risk of developing an SPM. For this group, we found that patients diagnosed with ASCC first were more likely to have later diagnoses of ​another primary tumor in the lung, oropharynx, vulva, or colon/rectum/anus. We identified these SPMs using histology and morphology groupings outlined in the coding rules for the SEER dataset as described in the Methods section above, and care was taken to avoid misclassification of primary recurrences of ASCC as SPMs.

In Cohort 2 (patients in whom ASCC was diagnosed as an SPM after another index cancer), the overall SIR for both men and women was 1.23, representing an overall increased relative risk of 23%. However, these SIR values were particularly enriched with regards to developing SPMs in specific organ systems. We observed that patients diagnosed with a separate first anal cancer, Kaposi sarcoma, or vulvar cancer had an elevated excess risk of later diagnosis with ASCC. Interestingly, we did not observe an increased excess risk for the second diagnosis of Kaposi sarcoma in patients first diagnosed with ASCC. This may be related to the success of antiretroviral therapy in HIV+patients, which may be the cause of decreasing incidence of Kaposi sarcoma.

The elevated rate of oral and pharyngeal cancers or vulvar cancers in patients with ASCC may be related to HPV infection status, an important consideration with these cases in particular.^10^, ^11^, ^15^ In patients with Kaposi sarcoma and ASCC, HIV infection has been thought to play a role in immunosuppression‐related tumor development in an indirect manner.^16^ HIV infection status is not reported within the SEER registry and therefore this relationship could not be explored within this study. We observed a link between ASCC and Kaposi sarcoma in men, but not in women.

Increased risk of cancers, particularly ASCC, with known infectious etiologies has been demonstrated in HIV+patients compared to HIV‐ patients.^17^ Other viral coinfections are common with HIV, including HPV and hepatitis B and/or C viruses. Additionally, HIV infection is associated with an increased risk of other non‐AIDS‐related cancers, the most common of which is lung cancer.^17^ In this study, we could not ascertain if any of the patients diagnosed with ASCC had underlying HIV or other viral infections predisposing them to other malignancies, whether due to a state of chronic immunosuppression or another mechanism. Additionally, we were unable to assess risk factors such as smoking or other exposures that may be common among patients who experience a cancer diagnosis.

The strongest association we discovered was the elevated rate of lung cancer diagnoses in patients previously diagnosed with ASCC. This is consistent with previous reports using older versions of the SEER registry as well as with institutional studies conducted on a smaller scale.^9^, ^11^, ^18^ Based on our present understanding, there is no clear mechanism for this relationship. Indeed, the explanation for this relationship is likely multifactorial: underlying viral co‐infection, genetic or epigenetic factors, treatment‐related immunosuppression, or other risk factors which may be similar in patient populations with anal cancer and lung cancer (e.g., smoking/tobacco use) which may contribute to this correlation.^5^ We did not observe an excess risk of ASCC diagnosis in patients first diagnosed with lung cancer. However, this could partially be due to a higher competing risk of death among patients with lung cancer when compared to patients with other more indolent cancer types. Detailed information about chemotherapy and/or radiation therapy, genomic analysis of the tumors, and particular patient information such as HIV or HPV status and smoking history are unavailable in this dataset and represents a relative limitation of this study.

We also examined the role of radiation therapy in ASCC treatment and whether it may be related to increased rates of SPMs. While overall the increased incidence for SPMs was independent of EBRT (including for lung cancer and second primary anal cancers), we found that radiation treatment of ASCC to the pelvis could be associated with increased risk of certain SPMs, including subsequent genital system cancers in female patients. Further investigation on this increased risk is warranted, especially related to HPV‐associated malignancies and the effect of radiation on the risk of their development in patients treated for ASCC. On the other hand, radiation for ASCC in male patients may serve a protective role for cancers such as prostate cancer. This observation is consistent with a previous report using data from a Netherlands population‐based cancer registry, which found that radiation therapy was protective against prostate cancer in patients treated for rectal cancer.^19^ This may be due to several reasons, that is, non‐target radiation dose to the prostate affecting occult malignancies, or other mechanisms such as testicular radiation dosage that might result in androgen‐driven changes affecting the development of prostate cancer. This appears to be an effect specific to prostate cancer, as we observed a similar excess risk of developing both pelvic (urinary bladder) and non‐pelvic (lymphatic/hematopoietic) SPMs in men treated with radiation therapy for ASCC.

In this study, we found that patients diagnosed with ASCC are often survivors of previous cancer or are at increased risk for developing another malignancy. These findings provide a basis for consideration of expanded surveillance and screening in patients diagnosed with anal squamous cell carcinoma, specifically for the development of lung, colon/rectal/anal, oropharyngeal, and vulvar cancers. In patients diagnosed with Kaposi sarcoma or vulvar cancer as an index case, screening or surveillance for anal cancer should be expanded as well. Furthermore, these findings highlight the need for ASCC survivorship plans to promote lifestyle changes associated with reduced cancer risk, such as tobacco and alcohol abstinence and physical activity as well as continued cancer screening measures.

## CONFLICT OF INTEREST

The authors of this study indicate no potential conflict of interest. Author disclosures are as follows: Dr. Jabbour receives grant funding from and is a research consultant for Merck, and a consultant for Syntactx. Dr. Romesser receives research support from and is a consultant for EMD Serono and is a board member of the HPV Alliance and Anal Cancer Foundation. Dr. Wu receives research grants from CivaTech Oncology, Inc., personal fees from AstraZeneca, and is on the advisory board of Simphotek, Inc. Dr. Jethwa receives honoraria from Radoncquestions.com, LLC.

## AUTHOR CONTRIBUTIONS

KSJ and SKJ conceptualized the project, wrote the original draft of the manuscript, and edited it. SKJ supervised and administered the project. KSJ collected data. KSJ and SEL analyzed data and wrote the methodology. SEL and JDM also analyzed data and wrote methodology and the original draft of the manuscript. PBR, KRJ, DL, AC, AJW, LH, and CH reviewed and edited the data, analysis, and manuscript.

## Data Availability

The data underlying this article are available through the SEER registry at seer.cancer.gov, reference number 12. The authors will also provide researchers with the full analyzed dataset upon request.
